# The psychometric properties of instruments measuring ethical sensitivity in nursing: a systematic review

**DOI:** 10.1186/s13643-024-02473-9

**Published:** 2024-11-20

**Authors:** Lu Zhou, LiXiong Bi, YuMing Wu, Lei Wang, Gao Liu, EnLi Cai

**Affiliations:** 1 School of Nursing, Yunnan University of Chinese Medicine, Kunming, China; 2 School of Medicine, Yunnan University of Chinese Medicine, Kunming, China

**Keywords:** Ethical sensitivity, Instruments, Nursing, Psychometric properties, Systematic review

## Abstract

**Background:**

Recognizing and appropriately responding to ethical considerations is a crucial element of ethical nursing practice. To mitigate instances of ethical incongruity in healthcare and to promote nurses’ comprehension of their professional ethical responsibilities, it is imperative for researchers to accurately evaluate ethical sensitivity. Conducting a systematic review of the available instruments would enable practitioners to determine the most suitable instrument for implementation in the field of nursing.

**Aim:**

This review aims to systematically assess the measurement properties of instruments used to measure ethical sensitivity in nursing.

**Methods:**

A systematic literature search was conducted in July 2022 in the following electronic databases: Scopus, CINAHL, APAPsycINFO, Embase, Web of Science, and PubMed. Two reviewers independently screened and assessed the studies in accordance with the COnsensus-based Standards for the selection of health Measurement INstruments (COSMIN) checklist. The updated criteria for good measurement properties are used to rate the result of measurement properties, and the modified Grading of Recommendations Assessment, Development and Evaluation (GRADE) approach was used to grade the quality of the summarized evidence.

**Results:**

This review encompasses a total of 29 studies that describe 11 different instruments. Neither cross-cultural validity nor responsiveness was examined in any of the included studies. Whereas the majority of the instruments were conducted with at least some type of validity assessment, nearly all of the reliability results rated were indeterminate. Two instruments were recommended, the Ethical Sensitivity Questionnaire for Nursing Students (ESQ-NS) and the Ethical Awareness Scale for nurses in intensive care units. It is recommended that new self-administration instruments for special nursing settings be developed in accordance with the item response theory (IRT)/Rasch model.

**Conclusion:**

The selection of ethical sensitivity measurement instruments in nursing, and further research on the development, psychometric, and cross-cultural adaptation of these instruments, could be conducted in accordance with the findings and suggestions of this systematic review.

**Strengths and limitations:**

• This review was conducted to assess 11 instruments that were used to measure ethical sensitivity in nursing in 29 studies.

• The Ethical Sensitivity Questionnaire for Nursing Students (ESQ-NS) and the Ethical Awareness Scale for nurses in intensive care units can be recommended, but further reliability and cross-cultural validity testing are needed.

• The IRT/Rasch model is also recommended to measure ethical sensitivity in nursing.

• The potential limitation of utilizing the COSMIN checklist for assessing methodological quality is worth considering.

• Test–retest was considered inappropriate; thus, the reliability testing of ethical sensitivity measurement instruments still needs to be explored.

**Supplementary Information:**

The online version contains supplementary material available at 10.1186/s13643-024-02473-9.

## Introduction

The complex ethical dilemmas arising from advancements in medicine and the increased demands of the healthcare industry are currently being come across [1, 2]. Medical professionals are required to possess a greater ethical awareness and responsibility; hence, the essential role of ethics in health professional education is on the rise [3, 4]. Ethical competence has been considered one of the professional components [5]. In addition, due to the unique culture of the healthcare profession, which encompasses its values [6], nurses who are responsible for coordinating the healthcare team and patients often encounter conflicting values, particularly ethical dilemmas [7–9]. In this process, nurses must improve ethical sensitivity to make ethically sound judgments in their application [10].

Ethical sensitivity has also been termed “moral sensitivity” in many studies of the concept [11]. Ethical sensitivity has been identified as a foundational component of ethical action according to Rest in 1976 [11, 12]. Subsequently, scholars have proposed various theories and models pertaining to ethical sensitivity. The prevalent model employed to cultivate and execute ethical sensitivity in nursing setting is the concept of moral sensitivity, which was developed by Lützén in 1993 [13]. Lützén defined moral sensitivity as “the ability to recognize a moral conflict” and “have insight into the ethical consequences made on behalf of the person.” In 2001, Ersoy and Goz [14] defined it as “the capacity or ability to recognize an ethical problem (or an ethical dimension when an ethical conflict is not present).” Weaver et al. [15] conceptualized ethical sensitivity in 2008 as well, i.e., that which enables professionals to recognize, interpret, and respond appropriately to the concerns of those receiving professional services. In their recently published concept analysis, Milliken et al. integrated the concept of ethical sensitivity by proposing that ethical awareness is a component of ethical sensitivity [11], which is the first step in the process of ethical action. Ethical sensitivity, in turn, is an important component of moral reasoning [16].

Ethical sensitivity is imperative in good (ethical) patient care [11] due to enhancing better ethical behavior [17]. However, evidence suggests the ethical import of everyday issues may go unnoticed by nurses in practice, putting patients at risk for harm, and nurses may, at times, feel underprepared to recognize and address ethical issues as they arise in practice [18, 19]. Moreover, diminished or absent ethical sensitivity can result in ethically incongruent care, which is inconsistent with the professional obligations of nursing. Social and technological developments, professional conflicts, and unawareness of individuals regarding their rights are known to create ethical dilemmas [20], and continuous exposure to ethical dilemmas in practice is associated with negative consequences for nurses, such as moral distress [21], which can also have adverse effects on patients, such as decreasing quality of patient care, decreasing confidence in nursing services, and prolonged hospital stay [22].

As such, identifying the most appropriate way to assess ethical sensitivity in nursing is imperative to design interventions to facilitate ethical practice and to ensure nurses recognize the nature and extent of professional ethical obligations. Although many instruments have been internationally developed to assess ethical sensitivity in nursing, it is still not easy to choose the most appropriate instrument for a specific purpose. In part, this is because a comprehensive summary of existing instruments and their measurement properties does not exist. A systematic review of measurement properties would effectively reveal which instruments have been tested and help researchers select an appropriate instrument [23]. Systematic reviews can provide a comprehensive overview of the measurement properties and support evidence-based recommendations in the selection of the most suitable instrument for a given purpose. These kinds of systematic reviews can also be conducted to identify gaps in knowledge regarding measurement properties, which can, in turn, be used to design new studies on measurement properties [24].

However, there are no studies that have conducted systematic reviews of measurement instruments of ethical sensitivity targeted to nursing groups. Although previous integrative reviews exist [11], these did not solely focus on the evaluation of psychometric properties. In-depth evaluations of all available reliability, validity, and responsiveness data for existing instruments used to measure ethical sensitivity in nursing have not been conducted, including assessing the methodological quality of studies and the psychometric properties and quality of evidence for measurement instruments.

Hence, this review aims to systematically identify and critically assess the psychometric properties of instruments used to measure ethical sensitivity in nursing [25].

## Methods

### Protocol and registration

This systematic review is based on PRISMA [26] (Preferred Reporting Items of Systematic Reviews and Meta-Analyses) reporting guidelines for project reporting. This systematic review has been registered in the PROSPERO [27] database (CRD42022325433).

### Literature search

A search was conducted in the following electronic databases: Scopus, the Cumulative Index of Nursing and Allied Health Literature (CINAHL), APAPsycINFO, Embase, Web of Science, and PubMed. The search was limited to publications in the English, Chinese, Korean, Japanese, and Turkish languages (coverage: inception to June 2022). In addition, the references of the included studies and identified reviews were examined, and a manual search was performed with the Google Scholar web search engine. The search strategy used Medical Subject Headings (MeSH) and keywords and included a combination of the following five aspects in reference to the search construct developed by Terwee et al. [28]:

- #1 construct search: "Ethical sensitivity"[tiab] OR “Moral sensitivity”[tiab] OR”Awareness”
- #2 population search: "Nurses"[Mesh] OR "Students, Nursing"[Mesh] OR "Nursing"[Mesh]
- #3 instruments search: Instrument[tiab] OR instruments[tiab] OR measure [tiab] OR measures[tiab] OR questionnaire [tiab] OR questionnaires[tiab] OR scale[tiab] OR scales[tiab] OR tool[tiab] OR tools[tiab] OR survey [tiab] OR test [tiab]
- #4 filter for measurement properties
- #5 exclusion filter

These filters were adapted for use when searching all the other databases and detailed in Supplementary material file 1 [28].

References identified by the search strategy were entered into EndNote bibliographic software to screen the selected articles [29].

### Eligibility criteria

Inclusion criteria are as follows: (a) original research; (b) pertains to an instrument designed to measure ethical sensitivity in nursing, with a focus on the development and evaluation of psychometric properties; and (c) published in English, Chinese, Korean, Japanese, and Turkish.

Exclusion criteria are as follows: (a) research that employed the instruments solely to validate other instruments measuring similar or related constructs was excluded, and (b) research that ethical sensitivity was only included as part of a broader measure were also excluded.

### Data selection

The search hits were inserted in EndNote, and duplicates were removed. Based on the established eligibility criteria for article selection, one author ran the reviewed retrieve strategy across all the databases, while another two independently screened the titles and abstracts. The search results were then screened with full-text review. Potential disagreements regarding the inclusion of an article were resolved through a discussion, but, in case of differences, a third researcher decided whether to include an article.

### The methodological quality and result rating of each single study

The COSMIN risk-of-bias checklist [25] is utilized for assessing the methodological quality and the psychometric properties of each single study. The list is composed of 10 boxes: instrument development, content validity, structural validity, internal consistency, cross-cultural validity, reliability (test–retest, inter-rater, intra-rater), measurement error, criterion validity, hypothesis testing, and responsiveness [30]; every box is evaluated with items assessed on a 4-point rating scale (very good, adequate, doubtful, inadequate), and the final evaluation of each property is assessed on a “worst score counts” principle.

The updated criteria for good measurement properties [24] is employed to rate the result of each single study on a measurement property. The term “quality” pertains to the actual outcome of the measured property. Each attribute is rated as sufficient ( +), insufficient (-), or indeterminate(?).

### Synthesis

The summary of evidence for each measurement property was conducted, and the quality of evidence for each property was graded as either “high,” “moderate,” “low,” or “very low” [24], in accordance with the modified Grading of Recommendations Assessment, Development and Evaluation (GRADE) quality of evidence method [24, 31]. The focus is here on each instrument. The results of all available studies on a measurement property are quantitatively pooled or qualitatively summarized and compared against the criteria for good measurement properties to determine whether overall the measurement property of the instrument is sufficient ( +), insufficient (-), inconsistent ( ±), or indeterminate (?) [24, 32].

The included instruments are categorized into three categories of recommendations: (A) instruments that have the potential to be recommended as the most suitable instrument for the construct and population of interest (i.e., instruments with evidence for sufficient content validity (any level) and at least low evidence for sufficient internal consistency); (B) instruments that may have the potential to be recommended, but further validation studies are needed (i.e., instruments categorized not in A or C); and (C) instruments that should not be recommended (i.e., instruments with high-quality evidence for an insufficient measurement property) [24, 33]. The step is formulated concerning the quality of the evidence, construct of interest, and study population.

The two authors conducted the aforementioned assessments autonomously, and the ultimate outcomes were attained via consensus.

## Results

### Study selection

**Fig. 1:**
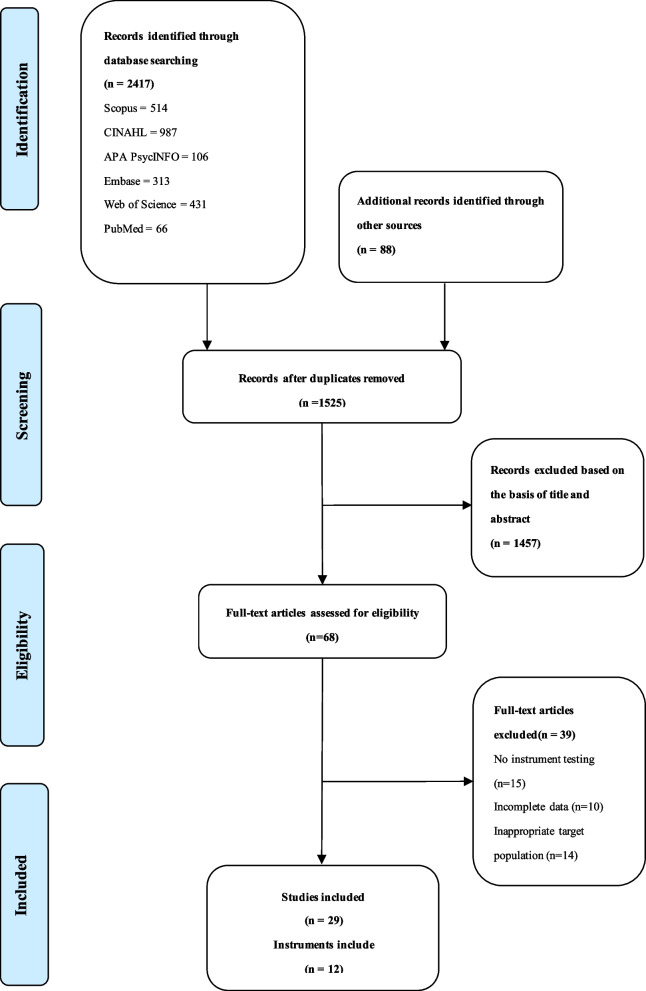
PRISMA literature screening flowchart

A total of 2505 articles were retrieved, 1525 duplicate articles were removed, and 980 articles were rescreened. Two reviewers evaluated the title and abstract based on the inclusion criteria. A total of 68 articles are included for full-text review. Among them, 29 studies met the search criteria shown in the PRISMA flowchart [26], as shown in Fig. 1.

### Studies and instruments characteristics

Most studies aimed at instrument development were conducted in the USA [34–37]. Sweden [38, 39], Spain [40, 41], and Japan [42, 43] have each developed two instruments. Fourteen studies aimed at instrument development and adaptation in 30 studies, while the remaining 16 studies focused on cross-cultural revisions and adaptations. A cross-sectional design with convenience sampling was employed in all 28 studies, while a few studies used purposive sampling [34, 36, 37, 40, 44] and random sampling [35, 45, 46]. The target population for each study was registered nurses or nursing students, with sample sizes ranging from 6 to 1465. Most of the study settings were hospitals or universities, but a few studies were conducted in primary healthcare centers [40, 41, 47]. Among the 12 instruments, 6 instruments were used to measure nurses’ ethical sensitivity across clinical settings, 4 instruments were targeted mainly at nursing students [34, 42, 43, 48], and the remaining 2 instruments were targeted at registered nurses in intensive care units [36] and primary health care professionals [41]. Two instruments were unidimensional models in accordance with the item response theory (IRT)/Rasch [36, 40]. There is one structured qualitative instrument [14]. The number of items in the measurement instruments ranged from 9 to 35. The most common instrument was the Moral Sensitivity Questionnaire (MSQ) [13]. The characteristics of the included studies and instruments are detailed in Table 1.

**Table 1:** Characteristics of included studies and instruments

Instrument	References	Language — country	Study aim(s)	According to	Setting	Design	Sampling	Population	Assessment	Instrument description
Original Moral Sensitivity Questionnaire (MSQ) 1994	Lützén et al. (1995) [38]	English — Sweden	OID/PT	Definition of moral sensitivity (Lützén, Johansson, and Nordström*)	Psychiatric clinics and medical-surgical clinics	Cross-sectional	Consecutive	145 psychiatric nurses & 150 surgical nurses	Self-reporting	30 items divided into 6 subscales: from 1 “completely in disagreement” to 7 “completely in agreement” 1. Interpersonal orientation 2. Structural moral meaning 3. Expressing benevolence 4. Modifying autonomy 5. Experiencing moral conflict 6. Relying on physician knowledge
	Kuilman, Jansen, Mulder, Middel, & Roodbol (2020) [49]	English — Netherlands	R/PT	Original Moral Sensitivity Questionnaire by Lützén (1994)	Hospitals	Cross-sectional	Convenience	155 nurse practitioners (NPs) & physician assistants (PAs)	Online Self-reporting	11 items divided into 2 subscales: from 1 “fully disagree” to 7 “fully agree” 1. Paternalistic attitude 2. Deliberate attitude
	Nora et al. (2018) [44]	Portuguese — Portugal	R/T	Original Moral Sensitivity Questionnaire by Lützén (1994)	Not stated	Cross-sectional	Purposeful	7 experts in the area of nursing, health or bioethics	N/A	28 items The rest characteristics of instrument were not stated
	Nora et al. (2018) [44]	Portuguese — Brazil	R/T	Original Moral Sensitivity Questionnaire by Lützén (1994)	Not stated	Cross-sectional	Purposeful	6 experts in the area of nursing, health, or bioethics	N/A	27 items divided into 4 subscales: from 1 “completely disagree” to 7 “completely agree” 1. Interpersonal orientation 2. Professional knowledge 3. Moral conflict 4. Moral meaning
	Dalla Nora, Zoboli, & Vieira (2019) [50]	Portuguese — Brazil	PT	Original Moral Sensitivity Questionnaire by Lützén (1994)	The health services of the Rio Grande do Sul	Cross-sectional	Convenience	316 nurses	Self-reporting	27 items divided into 4 subscales: from 1 “completely disagree” to 7 “completely agree” 1. Interpersonal orientation 2. Professional knowledge 3. Moral conflict 4. Moral meaning
	Nakamura, Ishikawa, & Hiejima (2000) [51]	Japanese — Japan	T/PT	Original Moral Sensitivity Questionnaire by Lützén (1994)	A university and its affiliated hospitals	Cross-sectional	Convenience	191 nursing and medical students	Self-reporting	35 items divided into 7 subscales: from 1 “completely in disagreement” to 6 “completely in agreement” 1. Responsibility of nurses and with respect of a patient 2. Faithfulness to judgment of a doctor and a rule 3. Introspection 4. Sincerity (a patient’s needs are accompanied) 5. Judgment of the care and conflict 6. Decision-making 7. Benevolence
	MichikoNAKAMURA et al. (2001) [52]	Japanese — Japan	T/PT	Original Moral Sensitivity Questionnaire by Lützén (1994)	Hospitals	Cross-sectional	Convenience	192 nurses	Self-reporting	35 items divided into 8 subscales: from 1 “completely in disagreement” to 6 “completely in agreement” 1. Role accomplishment 2. Respect for the patient’s spiritual integrity 3. Cordiality/conflict 4. Responsibility 5. Benevolence 6. Respect for the patient 7. Flexibility 8. Humanistic principles
	Han, Kim, Kim, & Ahn (2010) [53]	Korean — South Korea	T/PT	Original Moral Sensitivity Questionnaire by Lützén (1994)	A hospital	Cross-sectional	Convenience	283 nurses	Self-reporting	27 items divided into 5 subscales: from 1 “completely in disagreement” to 7 “completely in agreement” 1. Benevolence 2. Meaning 3. Conflict 4. Patient-oriented care 5. Professional responsibility
	Tosun (2018) [45]	Turkish — Türkiye	T/PT	Original Moral Sensitivity Questionnaire by Lützén (1994)	10 public hospitals	Cross-sectional	Cluster random	90 physicians & 90 nurses	Self-reporting	30 items divided into 6 subscales: from 1 “fully agree” to 7 “I do not agree at all” 1. Autonomy 2. Providing benefit 3. Holistic approach 4. Conflict 5. Application 6. Orientation
	Bayoumy, Halabi, & Esheaba (2017) [54]	Arabic — Kingdom of Saudi Arabia	T/PT	Original Moral Sensitivity Questionnaire by Lützén (1994)	The College of Nursing, affiliated with King Saud Bin Abdulaziz University for Health Sciences	Cross-sectional	Convenience	338 Saudi nursing students	Self-reporting	27 items divided into 7 subscales: rating not stated 1. Patient-centered care 2. Professional responsibility 3. Constructing moral meaning 4. Experience of moral dilemmas and conflicts 5. Nurse-patient relationship 6. Experience of good deeds 7. Guiding rules
Moral Sensitivity Questionnaire-2006	Lützen et al. (2006) [39]	English — Sweden	R/PT	Definition of moral sensitivity (Lützén, Johansson, & Nordström)	A health care conference & a hospital	Cross-sectional	Consecutive	123 health care conference participants & 155 physicians, nurses, and nursing assistants	Self-reporting	9 items divided into 3 subscales: from 1 “total disagreement” to 6 “total agreement” 1. Sense of moral burden 2. Moral strength 3. Moral responsibility
	Huang et al. (2016) [46]	Mandarin — China	T/PT	Moral Sensitivity Questionnaire by Lützén (2006)	Three tertiary regional referral centers (500þ beds each) and one municipal hospital (300–500 beds)	Cross-sectional	Cluster random	90 full-time registered nurses	Self-reporting	9 items divided into 2 subscales: from 1 “totally different” to 4 “equivalent” 1. Moral responsibility and strength 2. Sense of moral burden
	Maeda & Konishi (2012) [55]	Japanese — Japan	T/PT	Moral Sensitivity Questionnaire by Lützén (2006)	Hospitals	Cross-sectional	Consecutive	141 nurses	Self-reporting	9 items divided into 3 subscales: from 1 “total disagreement” to 6 “total agreement” 1. Sense of moral burden 2. Moral strength 3. Moral responsibility
	Jiménez-Herrera et al. (2022) [56]	Spanish — Spain	T/PT	Moral Sensitivity Questionnaire by Lützén (2006)	Four university campus	Cross-sectional	Consecutive	751 nursing students	Self-reporting	9 items divided into 3 subscales: from 1 “total disagreement” to 6 “total agreement” 1. Sense of moral burden 2. Moral strength 3. Moral responsibility
Modified Moral Sensitivity Questionnaire for Student Nurses (MMSQ-SN)	Comrie (2005) [34]	English — USA	R/PT	Definition of moral sensitivity (Lützén, Johansson, & Nordström)	Midwestern university school of nursing	Cross-sectional	Purposeful	250 nursing students	Self-reporting	30 items divided into 6 subscales: from 1 “strongly disagree” to 7 “strongly agree” 1. Interpersonal orientation 2. Structural moral meaning 3. Expressing benevolence 4. Modifying autonomy 5. Experiencing conflict 6. Professional Knowledge
	Yilmaz Sahin, Iyigun, & Acikel (2015) [57]	Turkish — Türkiye	T/PT	Modified Moral Sensitivity Questionnaire for Student Nurses by Comrie (2005)	A school of nursing in Ankara	Cross-sectional	Not stated	272 baccalaureate nursing students	Self-reporting	30 items divided into 6 subscales: from 1 “completely disagree” to 7 “completely agree” 1. Interpersonal orientation 2. Creating ethical meaning 3. Beneficence 4. Modifying autonomy 5. Experiencing the ethical dilemma 6. Getting expert opinion
Ethical Sensitivity Questionnaire for Nursing Students (ESQ-NS)	Muramatsu et al. (2019) [42]	Japanese/English — Japan	OID/PT	Definition of ethical sensitivity (Lützén, Johansson, & Nordström)	10 universities in the Chubu district belonging to the Japanese nursing-related university council	Cross-sectional	Convenience	525 nursing students	Self-reporting	13 items divided into 3 subscales: from 1 “totally different” to 4 “equivalent” 1. Respect for individuals 2. Distributive justice 3. Maintaining patients’ confidentiality
	Yu et al. (2021) [58]	Mandarin — China	T/PT	Ethical Sensitivity Questionnaire for Nursing Students (ESQ-NS) by Taeko Muramatsu (2019)	China Medical University, Liaoning University of Traditional Chinese Medicine, Shenyang Medical College, and Jinzhou Medical University	Cross-sectional	Convenience	1465 nursing students	Self-reporting	13 items divided into 4 subscales: from 1 “strongly disagree” to 4 “strongly agree” 1. Respect for individuals 2. Reasonable care 3. Distributive justice 4. Maintaining patients’ confidentiality
	Shengnan, Zhaobin, Pingping, & Xiumu (2022) [59]	Mandarin — China	T/PT	Ethical Sensitivity Questionnaire for Nursing Students (ESQ-NS) by Taeko Muramatsu (2019)	Universities in Bengbu city	Cross-sectional	Convenience	226 nursing students	Online Self-reporting	11 items divided into 3 subscales: from 1 “totally different” to 4 “equivalent” 1. Respect for individuals 2. Distributive justice 3. Maintaining patients’ confidentiality
	Min, Kim, & Lee (2020) [60]	Korean — South Korea	T/PT	Ethical Sensitivity Questionnaire for Nursing Students (ESQ-NS) by Taeko Muramatsu (2019)	Ewha Womans University	Cross-sectional	Convenience	138 nursing students	Online Self-reporting	13 items divided into 3 subscales: from 1 “strongly disagree” to 4 “strongly agree” 1. Critical understanding of the patient 2. Patient holistic care 3. Patient privacy and confidentiality
Moral sensitivity questionnaire for nursing students (MSQ-ST)	Takizawa et al. (2021) [43]	Japanese/English — Japan	R/PT	Definition of moral sensitivity (Lützén, Johansson, & Nordström)	25 nursing schools in the central area of Japan	Cross-sectional	Not stated	473 nursing students	Self-reporting	11 items divided into 3 subscales: from 1 “completely disagree” to 5 “completely agree” 1. Sense of moral burden 2. Moral strength 3. Moral responsibility
Byrd’s Nurse’s Ethical Sensitivity Test (Byrd’s NEST)	Byrd (2006) [35]	English — USA	OID/PT	Definition of moral sensitivity (Lützén, Johansson, & Nordström)	American Nurses Association (ANA) Affiliated Organizations	Cross-sectional	Random	520 registered nurses	Online Self-reporting	10 scenarios that cover the ethical dilemmas encountered during everyday nursing practices: from A “low” to C “high” Unclear whether the scale is unidimensional
Ethical Awareness Scale	Milliken et al. (2018) [36]	English — USA	OID/PT	Definition of ethical awareness: a component of ethical sensitivity, and the first step in the process of moral (or ethical) action (Milliken, 2016)	Intensive care units	Cross-sectional	Purposeful	127 registered nurses	Online Self-reporting	18 items: from 1 “never has ethical awareness” to 3 “always has ethical awareness” Unidimensional
	Milliken et al. (2019) [37]	English — USA	PT	Ethical Awareness Scale	Intensive Care Units	Cross-sectional	Purposeful	240 registered nurses	Online Self-reporting	18 items: from 1 “never has ethical awareness” to 3 “always has ethical awareness” Unidimensional
Ethical Sensitivity Scale for Clinical Nurses (ESSCN)	Joung & Seo (2020) [61]	Korean — South Korea	OID/PT	Definition of moral sensitivity (Lützén, Johansson, & Nordström)	Four general hospitals	Cross-sectional	Consecutive	344 nurses	Self-reporting	36 items divided into 8 subscales: from 1 “completely in disagreement” to 5 “completely in agreement” 1. Patient respect 2. Professional ethics 3. Nursing practice responsibilities 4. Willingness to do good 5. Ethical contemplation 6. Ethical burden 7. Perception of ethical situation 8. Empathy
Ethical Sensitivity Scale	González‐de Paz et al. (2012) [40]	Spanish — Spain	OID/PT	The concept of ethical sensitivity: that which enables professionals to recognize, interpret, and respond appropriately to the concerns of those receiving professional services (Weaver et al. 2008)	Hospitals & primary healthcare centers	Cross-sectional	Purposeful	159 nurses	Self-reporting	35 items: from 0 “I almost never consider the attitude” to 4 “I always consider the attitude” Unidimensional
Ethical attitudes questionnaire for PHC professionals	González-de Paz et al. (2013) [41]	Spanish — Spain	OID/PT	Normative ethics	Primary health care centers	Cross-sectional	Consecutive	273 professionals	Online self-reporting	24 general items divided into 5 subscales and 9 specific items: from 1 “never” to 5 “always” 1. Commitment to patients according to their healthcare needs 2. Communication between professionals and patients and professional protection 3. Use of information technology tools, continuing training, and use of computerized healthcare records 4. Self-identification of healthcare professional to patients 5. Patient decisions after professional communication
	González-de Paz et al. (2014) [47]	Spanish — Spain	PT	Ethical attitudes questionnaire for PHC professionals	56 primary health care centers	Cross-sectional	Consecutive	452 professionals	Online self-reporting	24 general items divided into 5 subscales: from 1 “never” to 5 “always” 1. Commitment to patients according to their healthcare needs 2. Communication between professionals and patients and professional protection 3. Use of information technology tools, continuing training and use of computerized healthcare records 4. Self-identification of healthcare professional to patients 5. Patient decisions after professional communication
Vignettes	Ersoy & Göz (2001) [14]	English — Türkiye	OID	International Council of Nurses (ICN) Code of Ethics	3 hospitals located in Kocaeli	Cross-sectional	Not stated	165 nurses	Self-reporting	4 hypothetical scenarios: provide written responses to the corresponding items based on the scenarios
Ethical Sensitivity Scale in Undergraduate Nursing Students (ESS-UNS)	Macale et al. (2015) [48]	English/Italian — Italy	OID/PT	Definition of ethical awareness: a milestone and a foundation regarding how to conduct actions in an ethical way	2 universities	Cross-sectional	Consecutive	517 baccalaureate nursing students	Self-reporting	20 items divided into 2 subscales: from 1 “maximum disagreement” to 5 “maximum agreement” 1. Ethical sensitivity as a positive attitude 2. Ethical sensitivity as a negative attitude

*OID* original instrument development. *R* revision. *T* translation. *PT* psychometric testing

^*^ “The ability to recognize a moral conflict” and “have insight into the ethical consequences made on behalf of the person” (Lützén, Johansson, & Nordström)

### The methodological quality of each single study

Instrument development was rated as doubtful or inadequate for 10 instruments in 12 studies due to a lack of evidence of data collection saturation, cognitive interviews, or pilot tests; only 2 studies were rated as adequate for methodological quality of instrument development [40, 41]. The content validity was assessed for 19 instruments in 29 studies, and doubtful data collection and analysis methods were the main reason for poor COSMIN scores, so that only 7 studies had adequate or very good content validity [36, 41, 46, 57, 59–61].

Construct validity was assessed using EFA in 19/26 classical test models, of which only 5 studies performed both EFA and CFA [43, 46, 56, 58, 59], but 1 study incorrectly selected the Rasch model [47]. The structural validity of the included classical test models was rated as inadequate or questionable due to inappropriate rotation methods and unclear CFA estimators (robust maximum likelihood (MLR)/diagonally weighted least squares (DWLS)). The methodological quality of the three IRT/Rasch models in terms of structural validity was rated adequate or very good [36, 37, 40]. The structural validity of the structured qualitative instrument was not performed.

Internal consistency was the most frequently assessed psychometric characteristic among the included studies and was of the best overall methodological quality, as most studies calculated internal consistency statistics for each unidimensional scale or subscale. Only 11 studies assessed reliability using test–retest methods [34, 35, 37, 39, 42, 45, 46, 55, 57, 58, 61], and all had time intervals greater than or equal to 2 weeks. Two studies did not use the retest method to measure reliability due to reflexivity, but did not mention other approaches to validation [34, 39].

The measurement invariance of the model was validated in 5 studies in 13 instrument development studies, but the methodological quality was rated as inadequate or doubtful due to insufficient sample size in the subgroup analysis [36, 37, 40, 42, 47] or inappropriate analysis methods (a linear regression analysis) [42]. None of the instruments for translation or cross-cultural revision validated the cross-cultural invariance of the model.

Measurement error was assessed in 1/29 studies [61], but COSMIN scores were rated as inadequate because it is unclear if SEM was calculated based on Cronbach’s alpha.

Criterion validity was assessed in the 5/29 study [35, 42, 58, 60, 61], using the MSQ and ESSCN as the gold standard.

Two studies that assessed convergent validity [43, 61] and 4/12 studies that assessed discriminative validity [40, 42, 48, 61] were rated as doubtful because only data presented on a comparison with an instrument that measures another construct or the statistical method applied was not optimal.

The responsiveness properties were not evaluated in any studies.

The results of the ratings for assessing measurement properties using the COSMIN risk-of-bias checklist are detailed in Table 2.

**Table 2:** COSMIN scores of the studies according to the number of psychometric tests

Instruments	References	Box 1				Box 2			Box 3	Box 4	Box 5	Box 6	Box 7	Box 8	Box 9		Box 10		
		1a.1	1a.2	1b	Total	2a	2b	Total							9a	9b	10a	10b	10c
MSQ-1994	Lützén et al. (1995) [38]	V	D	V	D	D	D	D	I	V									
	Kuilman et al. (2020) [49]	NA							A	V					A	A			
MSQ-P	Nora et al. (2018) [44]	NA				A	I	I											
MSQ-B	Nora et al. (2018) [44]	NA				A	I	I											
	Dalla Nora et al. (2019) [50]	NA							I	V									
MST-J	Nakamura et al. (2000) [51]	NA							D										
	MichikoNAKAMURA et al. (2001) [52]	NA							D										
K-MSQ	Han et al. (2010) [53]	NA				D	D	D	I	I									
ADA	Tosun (2018) [45]	NA				D	A	D		V		A							
A-MSQ	Bayoumy et al. (2017) [54]	NA				I	D	I	A	V									
MSQ-2006	Lützen et al. (2006) [39]	V	D	I	I				I			I							
MSQ-R-CV	Huang et al. (2016) [46]	NA				A	A	A	V	V		I							
J-MSQ	Maeda & Konishi (2012) [55]	NA				D	D	D	A	V		I							
MSQ-SPV	Jiménez-Herrera et al. (2022) [56]	NA							V	I						A			
MMSQ-SN	Comrie (2005) [34]	V	D	I	I	I		I		I		I							
The Turkish version of the MMSQSN	Yilmaz Sahin et al. (2015) [57]	NA				A	A	A		V		A							
ESQ-NS	Muramatsu et al. (2019) [42]	V	D	I	I	D	D	D	A	V	D	A		V		D			
The Chinese version of ESQ-NS	Yu et al. (2021) [58]	NA				I	A	I	D	V		A		V		A			
	Shengnan et al. (2022) [59]	NA				A	A	A	D	V						A			
KESQ-NS	Min et al. (2020) [60]	NA				A	A	A	I	V				V					
MSQ-ST	Takizawa et al. (2021) [43]	V	D	V	D	D	A	D	V	V					D	A			
Byrd’s NEST	Byrd (2006) [35]	V	D	A	D	D	D	D		D		A		V					
Ethical Awareness Scale	Milliken et al. (2018) [36]	V	I	I	I	V	V	V	V	V	I					V			
	Milliken et al. (2019) [37]	NA							V	V	I	A				V			
ESSCN	Joung & Seo (2020) [61]	V	D	D	D	V	V	V	I	V		A	D	V	D	D			
Ethical Sensitivity Scale	González‐de Paz et al. (2012) [40]	V	D	A	A				A	V	I					D			
Ethical attitudes questionnaire for PHC professionals	González-de Paz et al. (2013) [41]	V	A	A	A	V	V	V	I	V									
	González-de Paz et al. (2014) [47]	NA							I		I					V			
Vignettes	Ersoy & Göz (2001) [14]	V	D	D	D														
ESS-UNS	Macale et al. (2015) [48]			I	I				I	V						D			

Box 1, instruments development. 1a.1, design. 1a.2, concept elicitation (relevance and comprehensiveness). 1b, cognitive interview study or another pilot test. Box 2, content validity. 2a, asking targets. 2b, asking experts. Box 3, structural validity. Box 4, internal consistency. Box 5, measurement invariance/cross-cultural validity. Box 6, reliability. Box 7, measurement error. Box 8, criterion validity. Box 9, construct validity. 9a, convergent validity. 9b: Discriminative validity. Box 10, responsiveness. 10a, comparison with gold standard. 10b, comparison with other instruments. 10c, comparison between subgroups. I, inadequate; *D*, doubtful; *A* adequate; *V* very good. Empty boxes, not reported. *NA*, not applicable. Moral Sensitivity Questionnaire (*MSQ*), the Portugal version of Moral Sensitivity Questionnaire (*MSQ-P*), the Brazilian version of Moral Sensitivity Questionnaire (*MSQ-B*), the Japanese version of Moral Sensitivity Test (*MST-J*), the Korean version of the Moral Sensitivity Questionnaire(*K-MSQ*), Ahlaki Duyarlılık Anketi (*ADA*), the Arabic version of the Moral Sensitivity Questionnaire (*A-MSQ*), the Moral Sensitivity Questionnaire-Revised Chinese Version (MSQ-R-CV), the Japanese version of the Moral Sensitivity Questionnaire (J-MSQ), the Spanish version of the Moral Sensitivity Questionnaire (MSQ-SPV), Modified Moral Sensitivity Questionnaire for Student Nurses (MMSQ-SN), the Turkish Version of the Modified Moral Sensitivity Questionnaire for Student Nurses (The Turkish version of the MMSQSN), Ethical Sensitivity Questionnaire for Nursing Students (ESQ-NS), the Chinese version of Ethical Sensitivity Questionnaire for Nursing Students (the Chinese version of ESQ-NS), the Korean version of the Ethical Sensitivity Questionnaire for Nursing Students (KESQ-NS), Moral Sensitivity Questionnaire for Nursing Students (MSQ-ST), Byrd’s Nurse’s Ethical Sensitivity Test (Byrd’s NEST), Ethical Awareness Scale, Ethical Sensitivity Scale for Clinical Nurses (ESSCN), Ethical Sensitivity Scale in Undergraduate Nursing Students (ESS-UNS)

### The rating of results for instruments in each single study

Results of content validity for only three instruments [34, 41, 57] were rated as sufficient ( +) due to the fact that a clear description of reviewing instruments is lacking, or only target population was involved or doubtful design or method.

The structural validity of 3/26 classical test model studies [46, 54, 58] was rated as sufficient ( +), whereas the rest were mostly rated as insufficient (-) and indeterminate (?) due to not meeting model fit criteria, and factors explain < 50% of variance. There is 1/4 IRT/Rasch study [47] rated as insufficient (-) due to violation of unidimensionality.

Thirteen instruments with Cronbach’s alpha(s) < 0.70 for each unidimensional scale or subscale were therefore rated as insufficient (-).

Measurement invariance was rated as indeterminate (?) in 1/5 study [42] due to minimal important change (MIC) not being defined.

Reliability was assessed for 11 instruments, but the results of only 1 instrument were rated as sufficiently ( +) [61], benefiting from the fact that it reported intraclass correlation coefficient (ICC) and *ICC* > 0.70.

Measurement error results for ESSCN were rated indeterminate (?) because MIC and smallest detectable change (SDC) were not defined.

The correlation results of all 5 instruments [35, 42, 58, 60, 61] with the gold standard were < 0.70.

Only three classical test theory (CTT) models [43, 47, 49] and two Rasch models [36, 37, 40] defined specific hypotheses for construct validity, and the result is in accordance with the hypothesis, so the construct validity of the five instruments is sufficient ( +).

Using the updated criteria based on Terwee et al. [23] and Prinsen et al. [62] assessed the result of each single study on a measurement property as detailed in Table 3.

**Table 3:** Rating the measurement properties of the instruments according to the psychometric tests

Instruments	References	Relevance	Comprehensiveness	Comprehensibility	Content validity	Structural validity	Internal consistency	Measurement invariance/cross-cultural validity	Reliability	Measurement error	Criterion validity	Construct validity	Responsiveness
MSQ-1994	Lützén et al. (1995) [38]	+	-	+	-	-	-						
	Kuilman et al. (2020) [49]	-	-	-	-	?	+					+	
MSQ-P	Nora et al. (2018) [44]	+	?	?	?								
MSQ-B	Nora et al. (2018) [44]	+	?	?	?								
	Dalla Nora et al. (2019) [50]					?	-						
MST-J	Nakamura et al. (2000) [51]	-	-	-	-	?							
	MichikoNAKAMURA et al. (2001) [52]	-	-	-	-	?							
K-MSQ	Han et al. (2010) [53]	+	?	+	?	-	?						
ADA	Tosun, (2018) [45]	+	?	+	?		-		?				
A-MSQ	Bayoumy et al. (2017) [54]	?	?	?	?	+	-						
MSQ-2006	Lützen et al. (2006) [39]	?	-	+	-	?			?				
MSQ-R-CV	Huang et al. (2016) [46]	+	-	+	-	+	-		?				
J-MSQ	Maeda & Konishi (2012) [55]	?	?	+	?	-	-		?				
MSQ-SPV	Jiménez-Herrera et al. (2022) [56]	?	?	?	?	-	-					?	
MMSQ-SN	Comrie (2005) [34]	+	?	+	+		-		?				
The Turkish version of the MMSQSN	Yilmaz Sahin et al. (2015) [57]	+	+	+	+		+		?				
ESQ-NS	Muramatsu et al. (2019) [42]	+	?	+	?	?	+	?	?			?	
The Chinese version of ESQ-NS	Yu et al. (2021) [58]	+	?	+	?	+	+		?			?	
	Shengnan et al. (2022) [59]	+	?	+	?	-	+					?	
KESQ-NS	Min et al. (2020) [60]	+	?	+	?	-	+				-		
MSQ-ST	Takizawa et al. (2021) [43]	+	-	?	?	-	-					+	
Byrd’s NEST	Byrd (2006) [35]	+	?	?	?		-		-		-		
Ethical Awareness Scale	Milliken et al. (2018) [36]	+	?	+	?	+	+	+				+	
	Milliken et al. (2019) [37]					+	+	+	?			+	
ESSCN	Joung & Seo (2020) [61]	+	?	+	?	-	?		+	?	-	?	
Ethical Sensitivity Scale	González‐de Paz et al. (2012) [40]	+	?	+	?	+	-	+				+	
Ethical attitudes questionnaire for PHC professionals	González-de Paz et al. (2013) [41]	+	+	+	+	?	-						
	González-de Paz et al. (2014) [47]					-		+				+	
Vignettes	Ersoy & Göz (2001) [14]	+	?	?	?								
ESS-UNS	Macale et al. (2015) [48]	?	?	?	?	-	-					?	

Score: + sufficient. -Insufficient. ?Indeterminate. Empty boxes, not reported. Moral Sensitivity Questionnaire (MSQ), the Portugal version of Moral Sensitivity Questionnaire (MSQ-P), the Brazilian version of Moral Sensitivity Questionnaire (MSQ-B), the Japanese version of Moral Sensitivity Test (MST-J), the Korean version of the Moral Sensitivity Questionnaire(K-MSQ), Ahlaki Duyarlılık Anketi (ADA), the Arabic version of the Moral Sensitivity Questionnaire (A-MSQ), the Moral Sensitivity Questionnaire-Revised Chinese Version (MSQ-R-CV), the Japanese version of the Moral Sensitivity Questionnaire(J-MSQ), the Spanish version of the Moral Sensitivity Questionnaire(MSQ-SPV), Modified Moral Sensitivity Questionnaire for Student Nurses (MMSQ-SN), the Turkish Version of the Modified Moral Sensitivity Questionnaire for Student Nurses (the Turkish version of the MMSQSN), Ethical Sensitivity Questionnaire for Nursing Students (ESQ-NS), the Chinese version of Ethical Sensitivity Questionnaire for Nursing Students (The Chinese version of ESQ-NS), the Korean version of the Ethical Sensitivity Questionnaire for Nursing Students (KESQ-NS), Moral Sensitivity Questionnaire for Nursing Students (MSQ-ST), Byrd’s Nurse’s Ethical Sensitivity Test (Byrd’s NEST), Ethical Awareness Scale, Ethical Sensitivity Scale for Clinical Nurses (ESSCN), Ethical Sensitivity Scale in Undergraduate Nursing Students (ESS-UNS)

#### Synthesis

This systematic review has identified that six instruments were subjected to testing in multiple studies, while the remaining instruments were tested in only one study. The modified GRADE approach was applied to grade the quality of the evidence while summarizing and evaluating the measurement properties of these instruments in accordance with the criteria for good measurement properties. Additionally, categories of recommendations were formulated concerning the quality of the evidence, construct of interest, and study population. The results of the evidence synthesis are presented in Table 4.

- Moral Sensitivity Questionnaire (MSQ) (1994): The overall rating for content validity and reliability were indeterminate, so there will be no grading of the quality of the evidence [24]. Moderate quality for inconsistent internal consistency was found, since unexplained inconsistency of results across studies. The low quality of evidence showed inconsistent structural validity. However, sufficient construct validity was confirmed by the high quality of evidence. Thus, MSQ-1994 may have the potential to be recommended for various clinical settings, but further validation studies are needed.
- Moral Sensitivity Questionnaire (2006): The moderate and low quality of evidence showed inconsistency and insufficiency of content validity, structural validity, and internal consistency. Compared to MSQ-1994, it is shorter, so the recommended category is also B.
- Modified Moral Sensitivity Questionnaire for Student Nurses (MMSQ-SN): Except for internal consistency, which has been demonstrated to be inconsistent by moderate quality evidence, the remaining psychometric properties are uncertain and require further testing before the instrument can be recommended for measurement in nursing students.
- Ethical Sensitivity Questionnaire for Nursing Students (ESQ-NS): Structural validity and cross-cultural invariance are questionable, which also reduces reliability. Fortunately, there is no high-quality evidence for these insufficient measurement properties. In addition, the criterion validity and construct validity of the ESQ-NS need to be validated by further studies. However, it is undeniable that the ESQ-NS is currently the most preferred and most common instrument targeted at nursing students, as well as with evidence for sufficient content validity and high evidence for sufficient internal consistency. Thus, the recommended category is A.
- Ethical Awareness Scale also has the potential to be recommended as the most suitable instrument for the unidimensional model and registered nurses in intensive care units, as there is moderate and above evidence of sufficient and relatively comprehensive measurement properties, especially content validity and internal consistency. Furthermore, the Rasch model [63] is ideally suited to address some of the limitations that have been problematic in attempting to measure the related construct of ethical sensitivity [36].
- Ethical Sensitivity Scale: High-quality evidence demonstrated sufficient structural validity, measurement invariance, and construct validity. However, due to indeterminate structural validity and insufficient internal consistency of low-quality evidence, the recommended category for this instrument is B. The Rasch model of nurses’ ethical sensitivity may have the potential to be recommended for various clinical settings.
- Ethical attitudes questionnaire for PHC professionals needs to be further validated for structural validity and internal consistency. Noted that the CTT model violates unidimensionality when assessing structural validity using the Rasch model.
- Vignettes did not report any psychometric properties except for content validity, and the comprehensiveness of the instrument may be insufficient.
- Ethical Sensitivity Scale in Undergraduate Nursing Students (ESS-UNS): Low-quality evidence showed insufficient structural validity and internal consistency. Therefore, after refining sufficient measurement properties supported by high-quality evidence, the ESS-UNS has the potential to be recommended for measuring ethical sensitivity of nursing students.

**Table 4:** Summary of findings

	Synthesis		Overall rating	Quality of evidence	Recommendation
Moral Sensitivity Questionnaire (MSQ) (1994)	Content validity		?	NA	B
	Relevance		+		
	Comprehensiveness		?		
	Comprehensibility		±		
	Structural validity	Multidimensional scale (2/4/5/6/7 subscales) No CFA performed	±	Very low^abc^	
	Internal consistency	Cronbach’s alpha(s) ranging from .08 to 0.82	±	Moderate^b^	
	Reliability	Pearson correlation coefficient 0.98	?	NA	
	Construct validity	Hypothesis confirmed	+	High	
Moral Sensitivity Questionnaire (2006)	Content validity		±	Moderate^a^	B
	Relevance		?		
	Comprehensiveness		-		
	Comprehensibility		+		
	Structural validity	Multidimensional scale (2/3 subscales)	±	Low^ab^	
	Internal consistency	Cronbach’s alpha(s) ranging from 0.14 to 0.82	-	Moderate^b^	
	Reliability	Influence of reflection	?	NA	
	Construct validity	No hypothesis defined	?	NA	
Modified Moral Sensitivity Questionnaire for Student Nurses (MMSQ-SN)	Content validity		?	NA	B
	Relevance		+		
	Comprehensiveness		?		
	Comprehensibility		+		
	Internal consistency	Cronbach’s alpha(s) ranging from 0.64 to 0.82	±	Moderate^b^	
	Reliability	Pearson correlation coefficients ranging from 0.81 to 0.98	?	NA	
Ethical Sensitivity Questionnaire for Nursing Students (ESQ-NS)	Content validity		+	Low^a^	A
	Relevance		+		
	Comprehensiveness		+		
	Comprehensibility		+		
	Structural validity	Multidimensional scale (3/4 subscales)	±	Very low^a^	
	Internal consistency	Cronbach’s alpha(s) ranging from 0.71 to 0.82	+	High	
	Measurement invariance/cross-cultural validity	DIF/MGCFA not reported	?	Low^a^	
	Reliability	Pearson correlation coefficients/ICC ranging from 0.27 to 0.81	±	Low^ab^	
	Criterion validity	No high correlation with MSQ/ESSCN	-	Moderate^a^	
	Construct validity	No hypothesis defined	?	NA	
Moral sensitivity questionnaire for nursing students (MSQ-ST)	Content validity		?	NA	C
	Relevance		+		
	Comprehensiveness		-		
	Comprehensibility		?		
	Structural validity	Multidimensional scale (3 subscales)	-	High	
	Internal consistency	Cronbach’s alpha(s) ranging from 0.44 to 0.76	-	High	
	Construct validity	Hypothesis confirmed	+	High	
Byrd’s Nurse’s Ethical Sensitivity Test (Byrd’s NEST)	Content validity		?	NA	C
	Relevance		+		
	Comprehensiveness		?		
	Comprehensibility		?		
	Internal consistency	Cronbach’s alpha 0.64	-	Moderate^a^	
	Reliability	Pearson correlation coefficient 0.17	-	Moderate^a^	
	Criterion validity	No significant correlation with MSQ/MES	-	High	
Ethical Awareness Scale	Content validity		+	Moderate^a^	A
	Relevance		+		
	Comprehensiveness		+		
	Comprehensibility		+		
	Structural validity	Unidimensional scale	+	Moderate^a^	
	Internal consistency	Cronbach’s alpha(s) ranging from 0.83 to 0.86 The reliability coefficient for person 0.81 The reliability coefficient for item 0.98 Index of person separation 2.07 Index of item separation 6.86	+	High	
	Measurement invariance	Ordinary least-squares (OLS) regression analysis (R^2^ ranging from 0.94 to 0.95, *p* < .001)	+	Moderate^a^	
	Reliability	ICC not reported	?	NA	
	Construct validity	Hypothesis confirmed	+	Moderate^a^	
Ethical Sensitivity Scale for Clinical Nurses (ESSCN)	Content validity		+	Moderate^a^	C
	Relevance		+		
	Comprehensiveness		+		
	Comprehensibility		+		
	Structural validity	Multidimensional scale (eight subscales) No CFA performed	-	low^a^	
	Internal consistency	Cronbach’s alpha(s) ranging from 0.61 to 0.92	-	High	
	Reliability	*ICC* 0.94	+	High	
	Measurement error	SEM 3.19, MIC not defined	?	NA	
	Criterion validity	Correlation with *MSQ* 0.52	-	High	
	Construct validity	No hypothesis defined	?	NA	
Ethical Sensitivity Scale	Content validity		?	NA	B
	Relevance		+		
	Comprehensiveness		?		
	Comprehensibility		+		
	Structural validity	Unidimensional scale; PCA	+	High	
	Internal consistency	Index of person separation 0.90	-	Low^a^	
	Measurement invariance	No DIF for age, place of work, or knowledge of the code of conduct was detected for any item; RUMM2030 software	+	High	
	Construct validity	Hypothesis confirmed	+	Moderate^a^	
Ethical attitudes questionnaire for PHC professionals	Content validity		+	High	B
	Relevance		+		
	Comprehensiveness		+		
	Comprehensibility		+		
	Structural validity	Multidimensional scale (5 subscales) No CFA performed; the chosen model (Rasch) did not fit the research question	-	Low^ab^	
	Internal consistency	Cronbach’s alpha(s) ranging from 0.65 to 0.90	-	Moderate^a^	
	Measurement invariance	No DIF for gender, type of profession, and level of knowledge of the charter of rights was detected for any item	+	Moderate^a^	
	Construct validity	Hypothesis confirmed	+	Moderate^a^	
Vignettes	Content validity		?	Moderate^a^	B
	Relevance		+		
	Comprehensiveness		?		
	Comprehensibility		?		
Ethical Sensitivity Scale in Undergraduate Nursing Students (ESS-UNS)	Structural validity	Multidimensional scale (two subscales) No CFA performed	-	Low^a^	B
	Internal consistency	Cronbach’s alpha(s) ranging from 0.34 to 0.92	-	Low^a^	
	Construct validity	No hypothesis defined	?	NA	

Notes [30]: Quality of the results rated as + positive rating, ?indeterminate rating, ± inconsistent rating, -negative rating

*ICC* interclass correlation coefficient, *EFA* exploratory factor analysis, *CFA* confirmatory factor analysis, *SEM* standard error of measurement, *PCA* principal components analysis, *DIF* differential item functioning, *MGCFA* multigroup confirmatory factor analysis

^a^downgrading for risk of bias

^b^downgrading for inconsistency.

^c^downgrading for imprecision

^d^downgrading for indirectness

Due to high-quality evidence for insufficient structural validity and internal consistency, Moral Sensitivity Questionnaire for Nursing Students (MSQ-ST), Byrd’s Nurse’s Ethical Sensitivity Test (Byrd’s NEST), and Ethical Sensitivity Scale for Clinical Nurses (ESSCN) should not be recommended.

## Discussion

This review systematically assesses studies of the psychometric properties of instruments to measure ethical sensitivity in nursing, the methodological quality of the studies, the quality of the measurement properties, and the grading of the available evidence to formulate recommendations. Twenty-nine studies and 12 instruments based on a reflective model were included in this systematic review. No studies were found in which the cross-cultural validity or responsiveness of included instruments was tested. Only two instruments used Rasch analysis to test the psychometric properties of the newly developed instruments, respectively, Ethical Awareness Scale and Ethical Sensitivity Scale. According to the COSMIN scoring system, two instruments were recommended, Ethical Sensitivity Questionnaire for Nursing Students (ESQ-NS) and Ethical Awareness Scale for nurses in intensive care units.

Content validity and internal consistency were considered priority measurement properties [24, 64]. In this systematic review, only one of the included studies [41] could be given at least adequate ratings for the methodological quality and sufficient results of content validity (detailed in Tables 2 and 3). The main reason for the insufficient content validity of most instruments found in the results of this study is that the comprehensiveness of content validity was omitted, i.e., the method of data collection and whether data saturation was achieved were not clarified [24], and also due to the reason that the relevance, comprehensiveness, and comprehensibility are assigned the same weight when evaluating content validity [24, 30]. Furthermore, according to Terwee et al. [23], Prinsen et al. [62], and Gignac et al. [65], we suggest that future studies use a method combining Cronbach’s alpha and McDonald’s omega coefficient (Ω) for testing internal consistency of CTT models, as Cronbach’s alpha has been shown to have a bias in underestimating internal consistency.

For the structural validity of CTT models, EFA was the preferred method in the included studies, but several studies [8/26] [38, 39, 48, 50, 53, 54, 60, 61] utilized an inappropriate rotational method (i.e., orthogonal instead of oblique rotation). It violates hypotheses for the correlation of the reflective model. Moreover, EFA alone is inadequate [24, 66]. Unfortunately, a minority of the studies [5/26] [43, 46, 56, 58, 59] performed CFA, which is a shortcoming in the level of evidence for structural validity. In terms of CFA estimator, one of the included studies [59] used a robust maximum likelihood (MLR) method; none of the rest mentioned it. However, the recent literature suggested that the diagonally weighted least squares (DWLS) may perform uniformly better than MLR in factor loading estimates for ordinal observed variables [67] since the use of MLR assumes that the observed indicators follow a continuous and multivariate normal distribution.

Classical test theory remains the most commonly employed approach for assessing the psychometric properties of instruments, providing evidence of scale-level properties [68], and a total of 9 instruments and 25 studies based on the CTT were included in this study. In contrast, Rasch analysis, which compares samples and items on the same equal scale in a log-transformed format, can offer more thorough data and evidence regarding item- and person-level traits [69], thus compensating for two shortcomings of the CTT: (a) the Likert scale is not equidistant, and (b) the same items may have different meanings on the same scores [70]. Moreover, objective measurement is one of the characteristics of the Rasch model [69], which may be more helpful in distinguishing the degree of ethical sensitivity of different nursing groups. Note that while reporting measurement properties of an instrument based on the IRT, the unidimensionality must not be violated, and a sample size of at least 100 per group is required for measuring invariance [25], but none of the four Rasch model studies [36, 37, 40, 47] included in this study fully met the appropriate sample size.

Given that ethical sensitivity is a latent variable [11], it is challenging to develop a gold standard for measuring ethical sensitivity. Nevertheless, none of the five studies in which evaluated criterion validity was conducted had measurements that met the criterion. Therefore, criterion validity is insufficient.

Resilience Measurement Scale, Behavioral Control targeted at Preventing Harm (BCPH) scale, Ethics Advocacy Scale (EAS), Moral Disengagement Scale (MDS), and the level of moral reasoning (DIT-N2) were used to validate convergent validity of included instruments [49]. Note that there is a need for authors to describe in detail the comparator instruments as well as demonstrate their reliability and validity in the study population as failure to do so affects both internal and external validity [25, 71]. However, 3/3 studies that tested convergent validity did not report the specific psychometrics of the comparator instruments, and 1/3 study refer to the psychometrics of the comparator from another population which again is questionable [43, 71]. Further, without demonstrating the psychometric robustness of the comparator instrument and making specific hypotheses, it is not possible to judge its construct validity [71]. None of the studies formulates specific hypotheses in comparison between subgroups to test discriminative validity.

### Future directions, recommendations and advice

While the aim of several instruments included in this review suggests measuring only one specific property, in-depth profiling of the defined dimensions being measured and reporting comprehensive psychometric properties are essential for instrument selection [24]. Further, cross-cultural validity testing is needed for the recommended models [36, 42] as well as for potentially recommended models with good content validity and internal consistency. It is also important to continuously update statistical data analysis methods for measurement characteristics to reduce bias (e.g. [24, 67]).

The included instruments differ in terms of the complexity and integrity relating to their items and dimensions. It is necessary to give a clear and operational definition of the construct when selecting instruments [64]. There is no standardized definition of ethical sensitivity exists, and the included instruments measure the general concept of ethical sensitivity or its different components. Seven of all included instruments were developed in accordance with the construct of ethical sensitivity proposed by Lützén et al. [13]: one was developed in accordance with the component of ethical sensitivity-Ethical Awareness Scale [36], one was developed in accordance with the concept proposed by Ersoy and Goz [14], and another two instruments [40, 41] were constructed based on the concepts defined by Weaver [15]. Overall, all included instruments reported conceptual models, except for the ESS-UNS [48]. Future research is needed to constantly integrate and develop an operational definition of ethical sensitivity in nursing and its different components.

New technologies and medical progress increasingly affect nursing globally [72], such as artificial intelligence (AI), robotic medical systems, precision healthcare, and increasing dependence on telehealth and other virtual models of nursing, which may bring new ethical problem [73] including algorithmic bias and fairness, equity in access to technology, ethical implications of robotics, and automation. The development of instruments requires a meticulous focus on ethics, with integrating established and evolving ethical frameworks, fostering interdisciplinary collaboration, and implementing robust education programs for nurses. Focus on the changes in ethical norms, as well as on global standards and regulations, is also essential. These provide the foundational principles and guidelines for the development of instruments.

Significantly, given a clear definition of the construct of interest when selecting an appropriate instrument, particular attention should also be paid to whether the instrument development study was conducted in the target population and whether other studies were conducted to test the psychometric properties of the instrument on the target population [24]. The instruments recommended by this systematic review were targeted at nursing students — ESQ-NS [42] and nurses in intensive care units — Ethical Awareness Scale [36]; additional testing of the cross-cultural validity of the two instruments is warranted. Further validation of available instruments is needed for groups of nurses in general clinical settings. Given the adequate availability of instruments that can be used with the abovementioned groups, the development of new self-administration instruments is not suggested. Especially, the Ethical Sensitivity Scale [40], if its internal consistency and content validity can be additionally validated in future studies, may address the measurement limitations of the MSQ [13, 39] due to insufficient structural validity. Finally, if the items are based on nursing groups in other special settings (e.g., pediatrics, obstetrics and gynecology, operating room, infectious disease, geriatrics, psychiatry, hospice), the development of new self-administration instruments in accordance with IRT/Rasch is suggested.

Overall, ethical sensitivity is a multidimensional construct [74], posing a formidable challenge in its assessment of construct validity, primarily attributed to the absence of a universally acknowledged gold standard. Therefore, whether engaging in the development of novel instruments or undertaking cross-cultural adaptation research, explicit delineation of an ethical sensitivity framework, the precise formulation of the initial structural definition, and the articulate expression of various dimensions play vital roles in the initial phases of instrument development. Further related research needs to cover a more comprehensive range of ethical literature, policies, and regulations, and conduct qualitative research, such as grounded theory [75], to ensure that the emergent themes and the operational definitions are comprehensive and accurate.

### Limitations

The potential limitation of utilizing the COSMIN checklist for assessing methodological quality is worth considering [32] as the checklist came into effect in 2011 and was updated in 2018, and some of the instrument development and translation were performed before the publication of the checklist.

Previous work suggested that in instruments of this type of psychological construct, there are reliability limitations. Test–retest was considered inappropriate as the type of questions utilized may stimulate respondents to reflect on the topic, which, in turn, may lead to new perspectives or attitudes toward the topic, causing inconsistent responses in multiple testing situations. Furthermore, it is not considered that the correlations found in this type of study are stable over time. Thus, the reliability testing of ethical sensitivity measurement instruments still needs to be explored.

## Conclusions

The present study conducted systematic reviews of instruments measuring ethical sensitivity in nursing in a transparent and standardized way. The findings can contribute to the development of ethical sensitivity instruments in nursing with cross-cultural adaptation, as well as an evidence-based selection of these instruments.

## Supplementary Information

**Additional file 1: Supplementary material file 1: Table 1.** Search strategy.

## Data Availability

Supplementary material associated with this article can be found in the online version.
