# Supplementary material for: The psychometric properties of instruments measuring ethical sensitivity in nursing: a systematic review
Source: Syst Rev. 2024 Nov 20;13:87. doi: 10.1186/s13643-024-02473-9 (PMC11577582; doi:10.1186/s13643-024-02473-9)
Supplement: Supplementary file 1 — Additional file 1: Supplementary material file 1: Table 1. Search strategy. [file 13643_2024_2473_MOESM1_ESM.pdf]

Supplementary Material-File 1

The psychometric properties of instruments measuring ethical sensitivity in nursing: A systematic review

Lu Zhou.[1], ·LiXiong Bi.[1], YuMing Wu.[2], Lei Wang.[1],Gao Liu.[1], and EnLi Cai.[1]

1 School of Nursing Yunnan University of Chinese Medicine, China;

2 School of Medicine, Yunnan University of Chinese Medicine, China;

* Correspondence: EnLi Cai: caienliycm@sina.cn(EL.C.);

Lu Zhou: zhoulutg@gmail.com(L.Z.);

LiXiong Bi: lxbycm@sina.com(LX.B.);

YuMing Wu: yumingwuycm@sina.com(YM.W.);

Lei Wang: wanglieycm@sina.com (L.W.);

Gao Liu: liugaoycm@sina.com (G.L.)

EnLi Cai: caienliycm@sina.cn(EL.C.);

ORCID

Lu Zhou: https://orcid.org/0000-0002-8314-053X

Table 1 Search strategy

#1	Ethical sensitivity[Title/Abstract]) OR (Moral sensitivity[Title/Abstract])) OR (Ethical awareness[Title/Abstract]
#2	"Nurses"[Mesh]) OR "Students, Nursing"[Mesh]) OR "Nursing Staff"[Mesh]
#3	Instrument[tiab] OR instruments[tiab] OR measure [tiab] OR measures[tiab] OR questionnaire[tiab] OR questionnaires[tiab] OR scale[tiab] OR scales[tiab] OR tool[tiab] OR tools[tiab] OR survey [tiab] OR test [tiab]
#4	Instrumentation[Subheading] OR methods[Subheading] OR “Validation Studies”[Publication Type] OR “Comparative Study”[Publication Type] OR “psychometrics”[MeSH] OR psychometr* [tiab] OR clinimetr* [tw] OR clinometr* [tw] “outcome assessment (health care)”[MeSH] OR “outcome assessment”[tiab] OR “outcome measure* ”[tw] OR “observer variation”[MeSH] OR “observer variation”[tiab] OR “Health Status Indicators”[Mesh] OR “reproducibility of results”[MeSH] OR reproducib* [tiab] OR “discriminant analysis”[MeSH] OR reliab* [tiab] OR unreliab* [tiab] OR valid* [tiab] OR coefficient[tiab] OR homogeneity[tiab] OR homogeneous[tiab] OR “internal consistency”[tiab] OR (cronbach* [tiab] AND (alpha[tiab] OR alphas[tiab])) OR (item[tiab] AND (correlation* [tiab] OR selection* [tiab] OR reduction* [tiab])) OR agreement[tiab] OR precision[tiab] OR imprecision[tiab] OR “precise values”[tiab] OR test-retest[tiab] OR (test[tiab] AND retest[tiab]) OR (reliab* [tiab] AND (test[tiab] OR retest[tiab])) OR stability[tiab] OR interrater[tiab] OR inter-rater[tiab] OR intrarater[tiab] OR intra-rater[tiab] OR intertester[tiab] OR inter-tester[tiab] OR intratester[tiab] OR intra-tester[tiab] OR interobserver[tiab] OR inter-observer[tiab] OR intraobserver[tiab] OR intraobserver[tiab] OR intertechnician[tiab] OR inter-technician[tiab] OR intratechnician[tiab] OR intra-technician[tiab] OR interexaminer[tiab] OR inter-examiner[tiab] OR intraexaminer[tiab] OR intra-examiner[tiab] OR interassay[tiab] OR inter-assay[tiab] OR intraassay[tiab] OR intra assay[tiab] OR interindividual[tiab] OR inter-individual[tiab] OR intraindividual[tiab] OR intra-individual[tiab] OR interparticipant[tiab] OR inter-participant[tiab] OR intraparticipant[tiab] OR intra-participant[tiab] OR kappa[tiab] OR kappa’s[tiab] OR kappas[tiab] OR repeatab* [tiab] OR ((replicab* [tiab] OR repeated[tiab]) AND (measure[tiab] OR measures[tiab] OR findings[tiab] OR result[tiab] OR results[tiab] OR test[tiab] OR tests[tiab])) OR generaliza* [tiab] OR generalisa* [tiab] OR concordance[tiab] OR (intraclass[tiab] AND correlation* [tiab]) OR discriminative[tiab] OR “known group”[tiab] OR “factor analysis”[tiab] OR “factor analyses”[tiab] OR dimension* [tiab] OR subscale* [tiab] OR (multitrait[tiab] AND scaling[tiab] AND (analysis[tiab] OR analyses[tiab])) OR “item discriminant”[tiab] OR “interscale correlation* ”[tiab] OR error[tiab] OR errors[tiab] OR “individual variability”[tiab] OR (variability[tiab] AND (analysis[tiab] OR values[tiab])) OR (uncertainty[tiab] AND (measurement[tiab] OR measuring[tiab])) OR “standard error of measurement”[tiab] OR sensitiv* [tiab] OR responsive* [tiab] OR ((minimal[tiab] OR minimally[tiab] OR clinical[tiab] OR clinically[tiab]) AND (important[tiab] OR significant[tiab] OR detectable[tiab]) AND (change[tiab] OR difference[tiab])) OR (small* [tiab] AND (real[tiab] OR detectable[tiab]) AND (change[tiab] OR difference[tiab])) OR “meaningful change” [tiab] OR “ceiling effect”[tiab] OR “floor effect”[tiab] OR “Item response model”[tiab] OR IRT[tiab] OR Rasch[tiab] OR “Differential item functioning”[tiab] OR DIF[tiab] OR “computer adaptive testing”[tiab] OR “item bank”[tiab] OR “cross-cultural equivalence”[tiab]
#5	#1 AND #2 AND #3 AND #4
#6	“Protocol”[Publication Type] OR “addresses”[Publication Type] OR“biography”[Publication Type] OR “case reports”[Publication Type] OR“comment”[Publication Type] OR “editorial”[Publication Type] OR“congresses” [Publication Type] OR “consensus development conference”[Publication Type] OR “consensus development conference”[Publication Type] OR “practice guideline”[Publication Type]) OR “suffering from”[tiab] OR “animals”[MeSH]
#7	#5 NOT #6
#8	FILTER: Language (English, Chinese, Korean, Japanese and Turkish)

Search: ((((((((Ethical sensitivity[Title/Abstract]) OR (Moral sensitivity[Title/Abstract])) OR (Ethical awareness[Title/Abstract])) OR (Moral awareness[Title/Abstract])) AND ((("Nurses"[Mesh]) OR "Students, Nursing"[Mesh]) OR "Nursing Staff"[Mesh])) AND (Instrument[tiab] OR instruments[tiab] OR measure [tiab] OR measures[tiab] OR questionnaire[tiab] OR questionnaires[tiab] OR scale[tiab] OR scales[tiab] OR tool[tiab] OR tools[tiab] OR survey [tiab] OR test [tiab])) AND ((((instrumentation[sh] OR methods[sh] OR "Validation Studies"[pt] OR "Comparative Study"[pt] OR "psychometrics"[MeSH] OR psychometr*[tiab] OR clinimetr*[tw] OR clinometr*[tw] OR "outcome assessment (health care)"[MeSH] OR "outcome assessment"[tiab] OR "outcome measure*"[tw] OR "observer variation"[MeSH] OR "observer variation"[tiab] OR "Health Status Indicators"[Mesh] OR "reproducibility of results"[MeSH] OR reproducib*[tiab] OR "discriminant analysis"[MeSH] OR reliab*[tiab] OR unreliab*[tiab] OR valid*[tiab] OR "coefficient of variation"[tiab] OR coefficient[tiab] OR homogeneity[tiab] OR homogeneous[tiab] OR "internal consistency"[tiab] OR (cronbach*[tiab] AND (alpha[tiab] OR alphas[tiab])) OR (item[tiab] AND (correlation*[tiab] OR selection*[tiab] OR reduction*[tiab])) OR agreement[tw] OR precision[tw] OR imprecision[tw] OR "precise values"[tw] OR test-retest[tiab] OR (test[tiab] AND retest[tiab]) OR (reliab*[tiab] AND (test[tiab] OR retest[tiab])) OR stability[tiab] OR interrater[tiab] OR inter-rater[tiab] OR intrarater[tiab] OR intra-rater[tiab] OR intertester[tiab] OR inter-tester[tiab] OR intratester[tiab] OR intra-tester[tiab] OR interobserver[tiab] OR inter-observer[tiab] OR intraobserver[tiab] OR intra-observer[tiab] OR intertechnician[tiab] OR inter-technician[tiab] OR intratechnician[tiab] OR intra-technician[tiab] OR interexaminer[tiab] OR inter-examiner[tiab] OR intraexaminer[tiab] OR intra-examiner[tiab] OR interassay[tiab] OR inter-assay[tiab] OR intraassay[tiab] OR intra-assay[tiab] OR interindividual[tiab] OR inter-individual[tiab] OR intraindividual[tiab] OR intra-individual[tiab] OR interparticipant[tiab] OR inter-participant[tiab] OR intraparticipant[tiab] OR intra-participant[tiab] OR kappa[tiab] OR kappa's[tiab] OR kappas[tiab] OR repeatab*[tw] OR ((replicab*[tw] OR repeated[tw]) AND (measure[tw] OR measures[tw] OR findings[tw] OR result[tw] OR results[tw] OR test[tw] OR tests[tw])) OR generaliza*[tiab] OR generalisa*[tiab] OR concordance[tiab] OR (intraclass[tiab] AND correlation*[tiab]) OR discriminative[tiab] OR "known group"[tiab] OR "factor analysis"[tiab] OR "factor analyses"[tiab] OR "factor structure"[tiab] OR "factor structures"[tiab] OR dimension*[tiab] OR subscale*[tiab] OR (multitrait[tiab] AND scaling[tiab] AND (analysis[tiab] OR analyses[tiab])) OR "item discriminant"[tiab] OR "interscale correlation*"[tiab] OR error[tiab] OR errors[tiab] OR "individual variability"[tiab] OR "interval variability"[tiab] OR "rate variability"[tiab] OR (variability[tiab] AND (analysis[tiab] OR values[tiab])) OR (uncertainty[tiab] AND (measurement[tiab] OR measuring[tiab])) OR "standard error of measurement"[tiab] OR sensitiv*[tiab] OR responsive*[tiab] OR (limit[tiab] AND detection[tiab]) OR "minimal detectable concentration"[tiab] OR interpretab*[tiab] OR ((minimal[tiab] OR minimally[tiab] OR clinical[tiab] OR clinically[tiab]) AND (important[tiab] OR significant[tiab] OR detectable[tiab]) AND (change[tiab] OR difference[tiab])) OR (small*[tiab] AND (real[tiab] OR detectable[tiab]) AND (change[tiab] OR difference[tiab])) OR "meaningful change"[tiab] OR "ceiling effect"[tiab] OR "floor effect"[tiab] OR "Item response model"[tiab] OR IRT[tiab] OR Rasch[tiab] OR "Differential item functioning"[tiab] OR DIF[tiab] OR "computer adaptive testing"[tiab] OR "item bank"[tiab] OR "cross-cultural equivalence"[tiab]))))))) NOT ((("addresses"[Publication Type] OR "biography"[Publication Type] OR "case reports"[Publication Type] OR "comment"[Publication Type] OR "directory"[Publication Type] OR "editorial"[Publication Type] OR "festschrift"[Publication Type] OR "interview"[Publication Type] OR "lectures"[Publication Type] OR "legal cases"[Publication Type] OR "legislation"[Publication Type] OR "letter"[Publication Type] OR "news"[Publication Type] OR "newspaper article"[Publication Type] OR "patient education handout"[Publication Type] OR "popular works"[Publication Type] OR "congresses"[Publication Type] OR "consensus development conference"[Publication Type] OR "consensus development conference, nih"[Publication Type] OR "practice guideline"[Publication Type]) NOT ("animals"[MeSH Terms] NOT "humans"[MeSH Terms])))) AND (((((English[Language]) OR (Chinese[Language])) OR (Korean[Language])) OR (Japanese[Language])) OR (Turkish[Language]))
